# Carious status and supragingival plaque microbiota in hemodialysis patients

**DOI:** 10.1371/journal.pone.0204674

**Published:** 2018-10-09

**Authors:** Qi Yue, Fei-Ting Yin, Qian Zhang, Chao Yuan, Mei-Yan Ye, Xiao-Ling Wang, Ji-Jun Li, Ye-Hua Gan

**Affiliations:** 1 Central Laboratory, Peking University School and Hospital of Stomatology & National Engineering Laboratory for Digital and Material Technology of Stomatology & Beijing Key Laboratory of Digital Stomatology, Beijing, China; 2 Department of Nephrology, the First Affiliated Hospital of General Hospital of PLA, Beijing, China; 3 Department of Preventive Dentistry, Peking University School and Hospital of Stomatology & National Engineering Laboratory for Digital and Material Technology of Stomatology & Beijing Key Laboratory of Digital Stomatology, Beijing, China; University Lyon 1 Faculty of Dental Medicine, FRANCE

## Abstract

**Objective:**

The aim of this study was to evaluate the carious status and the microbial profiles of supragingival plaque in patients with chronic kidney disease undergoing hemodialysis.

**Methods:**

This study included 30 patients with chronic kidney disease undergoing hemodialysis as well as 30 control subjects. Dental examination was performed and the decayed-missing-filled-teeth was recorded. Supragingival plaque was taken and analyzed using 16S rRNA gene amplicon by Illumina MiSeq sequencing to detect microbial composition and community diversity and structure.

**Results:**

The level of decayed-missing-filled-teeth was higher in the hemodialysis group than that in the control group. Microbial analysis showed a decrease in α diversity and a increase in relative abundance and prevalence of many acidogenic and aciduric caries related species in the supragingival plaque samples of the hemodialysis patients, including *Streptococcus mutans*, *Lactobacillus salivarius*, *Lactobacillus fermentum*, *Lactobacillus vaginalis*, *Scardovia wiggsiae F0424*, and *Actinomyces naeslundii*.

**Conclusion:**

Our results suggested that the hemodialysis patients were more susceptible to caries. More attentions for caries prevention and treatment should be paid to improve their life quality, and even to reduce their cardiovascular events and survival.

## Introduction

The association of oral diseases and systemic health is well documented [1–3]. Studies showed that oral diseases, including caries, periodontitis and mucosa diseases, etc., are highly prevalent and usually severe in CKD patients due to the malnutrition and uremia systemic changes[4]. In turn, oral diseases also have great influence on CKD prognosis. A very recent multinational cohort study demonstrated that in CKD hemodialysis patients, poorer dental health (higher carious status) is associated with early all-cause mortality, whereas preventive dental health practices are associated with longer survival [5]. These findings indicate that dental health may be a potentially preventative determinant of clinical outcomes in end-stage CKD patients. However, the carious status in CKD hemodialysis patients remains controversial, as both increased caries and decreased caries were reported in CKD hemodialysis patients [6–8]. There should be more CKD patients in China due to its huge population size, but only one study provided some oral status data from the Chinese CKD population [9].

The microbiome plays a key role in the etiology of many diseases. Dental caries is one of the most prevalent infectious oral diseases, which is mostly related with the bacterial shifts in supragingival dental plaque [10]. Oral microbiota imbalance results from changes of oral environment. Hemodialysis patients are usually in a complex condition in the end-stage of CKD. The uremia status and metabolic acidosis and also the treatments (medication and hemodialysis) may potentially alter the oral ecosystem, and affect oral microbiome, resulting in the soft and hard tissue diseases, such as periodontitis and caries. Data for microbiome changes associated with caries in hemodialysis CKD patients remains rare, and is also inconsistent while using the traditional cultural and the polymerase chain reaction (PCR) strategy. For instance, Al-Nowaiser et al. found that *Streptococcus mutans* decreases in CKD children [11], whereas Takeuchi et al. found that *Streptococcus mutans* and *Lactobacilli* increase in CKD adult patients [12]. The oral microbiome is highly diverse and complex, and dental caries is caused by bacterial community structure shifts rather than specific pathogenic bacteria [13]. It is proposed that the full spectrum of both culturable and non-culturable oral bacteria involved in disease should be considered [14]. Therefore, it remains to be explored by the recent technique of 16S rRNA gene sequencing whether there was bacterial shifts of supragingival plaque in the hemodialysis CKD patients.

Hemodialysis is associated with significant excess mortality and high social costs. Understanding the carious status, especially the early changes of caries related species in hemodialysis patients is very important for prevention or early intervention to improve life quality and survival of CKD patients than the general population [5]. Therefore, the aim of this study was to evaluate the carious status in hemodialysis patients, and to determine by 16S rRNA gene sequencing based on Illumina MiSeq platform whether there was any community structure change and caries-related shift of supragingival plaque microbiota.

## Materials and methods

### Subjects

Thirty CKD patients undergoing hemodialysis were recruited from totally 210 CKD patients in the Department of Nephrology, the First Affiliated Hospital of General Hospital of People’s Liberation Army. The CKD subjects were chosen using a simple random sampling method according to the inclusion criteria, and the individuals with serious physical or psychological illness or disadvantages and those who were unable or unwilling to attend the study were excluded. Therefore, the samples, to some extent, were representative of CKD hemodialysis patients. The inclusion criteria were as follows: age 18–60 years old; time on hemodialysis more than 12 months; glomerular filtration rate < 15 mL/min/1.73 m^2^; having more than 15 natural teeth (not including the crowned teeth, bridge abutments, pontics fixed dentures, removable partial dentures). The exclusion criteria were as follows: having diabetes; having acute oral disease; having received antibiotic treatment in the last 30 days; taking immunosuppressive drugs. The systemically healthy subjects as control group were recruited from the non-clinical staff of Peking University School and Hospital of Stomatology, and totally thirty systemically healthy subjects were included. The inclusion criteria were as follows: age 18–60 years old; having no systemic diseases and general medication use; glomerular filtration rate ≥ 90 mL/min/1.73 m^2^; having more than 15 natural teeth. The exclusion criteria were as follows: having acute oral disease; having received antibiotic treatment in the last 30 days. The control subjects and the CKD patients were matched at age, gender and smoking habits. Glomerular filtration rate was estimated from serum creatinine, using the four variables MDRD (modification of diet in renal disease) study equation [15]. This study obtained ethical approval of the Ethics Committee of Peking University Health Science Center (PKUSSIRB-201627036). Written informed consent from each subject was obtained in accordance with the Declaration of Helsinki.

### Medical and dental examination

All participants received medical evaluation, which consisted of demographic data and physical and laboratory tests of blood (creatinine, uric acid, urea, hemoglobin, glucose, C-reactive protein). The clinical characteristics included age, sex and duration on hemodialysis. The dental examinations were performed by a single calibrated experienced dentist from the Department of Preventive Dentistry, Peking University Hospital of Stomatology. This dental examiner was thoroughly trained in the Fourth National Oral Health Survey in China, and exhibited high level of intra examiner reliability. The number of decayed-teeth (DT), missing-teeth (MT), filled-teeth (FT), and the sum number of decayed-missing-filled-teeth (DMFT), an index for the general experience of caries, were recorded according to the World Health Organization guidelines for oral health surveys[16]. The plaque index representing oral hygiene condition was evaluated by Silness-Löe plaque index [17]. Briefly, the plaque scores from the four surfaces (buccal, lingual, mesial and distal) of six index teeth per subject were recorded and averaged for the group. Information on oral hygiene habits (tooth brushing, additional oral hygiene aids) and dietary habits (the times of sugary snacks and beverages per day) were obtained by a questionnaire.

### Supragingival plaque and saliva sampling

All participants were instructed not to clean their teeth overnight for 24 h and not to eat and drink 2 h before sample collection in the morning. For CKD patients, the supragingival plaque samples were collected bedside during one regular hemodialysis treatment time, and for the control, the plaque collection was performed on an appointment day. Supragingival plaque was pooled into a 1.5-mL sterile centrifuge tube (containing 1mL phosphate buffered saline) on ice after collected from buccal, lingual and interproximal surfaces of all the natural teeth of each subject using an individual sterile dental excavator, and then were stored at -80°C until microbial analysis. Non-stimulated saliva with a minimum of 2 mL was collected into a 5-mL sterile centrifuge tube on ice for biochemistry analysis.

The concentration of salivary urea and calcium were determined by automatic biochemistry instrument (Beckman AU 5800, USA). The saliva pH was measured with a pH strip (Spezialindikator, Merck, Darmstadt, Germany) according to Carlén, et al. [18]. The color change is then compared to a reference according to the manufacturer’s instructions. The examiners were trained in using the pH strips before the measurement commenced.

### DNA extraction

Bacteria genomic DNA of supragingival plaque specimens was isolated using a QIAamp DNA mini Kit (Qiagen, Hilden, Germany). The procedure was performed according to the manufacturer’s instructions with some modification, which the bacterial cell lysis was treated with lysozyme (20 mg/mL) for 1 h before the standard procedure [19, 20]. The elution volume was 50μL. The quantity and quality of DNA were evaluated by Nanodrop 8000 (Thermo, USA) and 1% agarose gel electrophoresis, respectively. All DNA of high-quality (the concentration ≥ 50 ng/μL, OD_260_/OD_280_ = 1.8–2.0) were stored at -20°C before further analysis.

### 16S rRNA sequencing

Polymerase chain reaction (PCR) for amplification of the V3-V4 region of bacterial 16S rRNA gene was performed using primers 338F (5’-GTACTCCTACGGGAGGCAGCA-3’) and 806R (5’-GTGGACTACHVGGGTWTCTAAT-3’) incorporating a sample barcode sequences. The PCR products were separated by 1% agarose gel electrophoresis and purified by Agencourt AMPure XP (Beckman Coulter, Inc., CA, USA). All the purified PCR products were pooled together to construct libraries according to the instructions and to be sequenced on the Illumina MiSeq PE300 sequencing platform (Illumina, Inc., CA, USA) by Beijing Allwegene Tech (Beijing, China).

### Sequencing analysis

Raw sequencing data were processed using the pipeline tools QIIME and MOTHUR. The sequences were separated from the sample barcode. Then, to obtain high-quality sequences for the downstream analysis, sequences less than 100 bp in length after splicing, contained one or more ambiguous base-calls (N), or less than 90% of quality scores Q20 were eliminated. Before further analysis, singleton operational taxonomical unit (OTU) was removed. After trimming, high-quality sequences were clustered into OTU using QIIME at a 97% similarity level. Each OTU was assigned taxonomically against the SILVA Ribosomal RNA databases [21]. Alpha diversity (Chao1, Observed species and Shannon) indexes were compared based on the least sequences (18487) and β diversity was determined by weighted UniFrac distance values. Principal component analysis (PCA) was conducted based on OTUs with different relative abundances.

### Quantification of bacterial load of *Streptococcus mutans*

Bacterial load of *Streptococcus mutans* was determined via real-time PCR using the species-specific primers (Forward 5’-TCGCGAAAAAGATAAACAAACA-3’/Reverse 5’-GCCCCTTCACAGTTGGTTAG -3’) [22]. 16S rRNA gene of 8 supragingival plaque samples (randomly selected samples that contained *Streptococcus mutans* according to 16S sequencing results, 4 samples from hemodialysis patients and 4 samples from controls) were amplified using universal primers 27F (5’-AGAGTTTGATCMTGGCTCAG-3’) and 1492R (5’-TACGGYTACCTTGTTACGACTT-3’). The PCR products were separated by 1% agarose gel electrophoresis and purified with Axyprep DNA gel extraction kit (Axygen, USA). The purified DNA were adjusted to 50 ng/μL, which were served as the templates. The standard curve was built from the PCR amplicons in triplicate of the *Streptococcus mutans* UA159 at diluted series concentrations (50 ng/μL, 5 ng/μL, 0.5 ng/μL, 0.05 ng/μL, 0.005 ng/μL, and 0.0005 ng/μL). All samples were amplified in triplicate to eliminate variation among tubes with the same templates. The amount of *Streptococcus mutans* was presented as gene copy number according to Novak et al. [23].

### Statistical analysis

Clinical and demographic data were compared via *t*-test, Chi-square or Wilcoxon Rank Sum test for parametric and non-parametric data, as appropriate using Statistical Package for Social Sciences (SPSS, version 20.0, Chicago, IL, USA). The normality of the data was verified using statistical analysis software SPSS 20. If the sig. value is more than 0.05 in Shapiro-Wilk test, the data was considered to obey the normal distribution. The non-normal distribution data was analyzed by nonparametric test. Differences in α diversity were evaluated by *t*-tests. Significant separation of clusters after PCA was evaluated via Analysis of Molecular Variance (AMOVA), as implemented in Mothur. Differences in relative abundances of individual taxa were determined via Wilcoxon Rank Sum test, while differences in taxon prevalence were tested via Chi-square test. At species level, LEfSe (version 1.0) was used to explore key biomarker in the two groups with the threshold on logarithmic linear discriminant analysis (LDA) score set to 2.0. The level of statistical significance was set at 0.05.

### Data access

All raw sequences were deposited in the NCBI Sequence Read Archive under accession number SRP126901.

## Results

### CKD hemodialysis patients showed higher dental caries than healthy controls

The demographics, biochemical characteristics and dental examination were shown in Table 1. There was no statistical difference in age, gender and smoking habits between the CKD hemodialysis group and the healthy control group. The DMFT and DT scores were both significantly higher in the CKD hemodialysis group than in the healthy control group (*P* < 0.01). The salivary urea and pH were also both significantly higher in the CKD hemodialysis group (*P* < 0.01), whereas the salivary calcium was significantly lower in the CKD hemodialysis group, as compared that in the healthy control group (*P* < 0.01).

**Table 1:** Demographics, blood and salivary tests and carious status of the participants.

	CKD (n = 30)	HC (n = 30)	*P*-value
Age (year)	48.53 ± 12.69	46.50 ± 8.83	0.475
Sex (male/female)	15/15	14/16	0.796
Smoker (yes/no)	8/22	9/21	0.774
Time on hemodialysis (month)	68.77 ± 46.70	—-	
Blood			
Creatinine (μmol/L)	1041.76 ± 216.93	76.68 ± 12.73	<0.01
Uric acid (μmol/L)	430.54 ± 64.98	308.37 ± 86.78	<0.01
Urea (mmol/L)	29.36 ± 6.96	4.86 ± 1.19	<0.01
Hemoglobin (g/L)	118.07 ± 11.64	144.43 ± 16.88	<0.01
Glucose (mmol/L)	5.33 ± 1.09	5.12 ± 0.68	0.398
C-reactive protein (mg/L)	3.09 ± 5.15	2.36 ± 3.89	0.547
Saliva			
Urea (mmol/L)	20.24 ± 7.73	5.83 ± 2.19	< 0.01
Calcium (mmol/L)	0.88 ± 0.30	1.27 ± 0.37	< 0.01
pH	8.21 ± 0.44	7.61 ± 0.35	< 0.01
Caries and oral hygiene			
DT	1.11 ± 1.62	0.10 ± 0.31	0.003
MT	2.18 ± 2.89	1.31 ± 2.17	0.205
FT	1.07 ± 1.72	0.86 ± 1.79	0.654
DMFT	4.36 ± 3.92	2.28 ± 2.52	0.022
PI	2.13 ± 0.45	1.98 ± 0.39	0.211

Values are expressed as mean ± SD or patient number; DT : decayed teeth; MT : missing teeth; FT : filled teeth; DMFT : decayed-missing-filled teeth; PI : plaque index; CKD: chronic kidney disease, HC: healthy control.

### CKD hemodialysis patients consumed sugar more frequently than healthy controls

The results of the questionnaire were presented in S1 Table. The frequency taking sugary snacks or beverages in the CKD hemodialysis group was significantly higher (*P* < 0.05). Moreover, the frequency (sometimes & always) of CKD hemodialysis patients experienced dry mouth significantly more than the healthy controls (*P* < 0.05). Both groups showed similar oral hygiene habits on brushing teeth, using toothpick, dental floss and mouthwash (*P* > 0.05).

### CKD hemodialysis patients showed different microbiota diversity from healthy controls

The bacterial genomic DNA was extracted from 58 supragingival plaques, and used for 16S rRNA gene amplification and sequencing. On average, 25289 ± 6150 filtered sequences and 156 ± 36 OTUs were generated per sample. The rarefaction curves approached asymptotes for most samples (S1 Fig). In total, 16 phyla, 28 class, 45 order, 67 family, 120 genera, and 121 species were detected in the supragingival plaque samples. At the phylum level, the vast majority of the sequences (> 90%) belonged to one of the five phyla (S2 Fig): *Proteobacteria*, *Firmicutes*, *Bacteroidetes*, *Fusobacteria*, and *Actinobacteria*, which were similar to that in a previous study with Chinese population [24].

**Fig 1:**
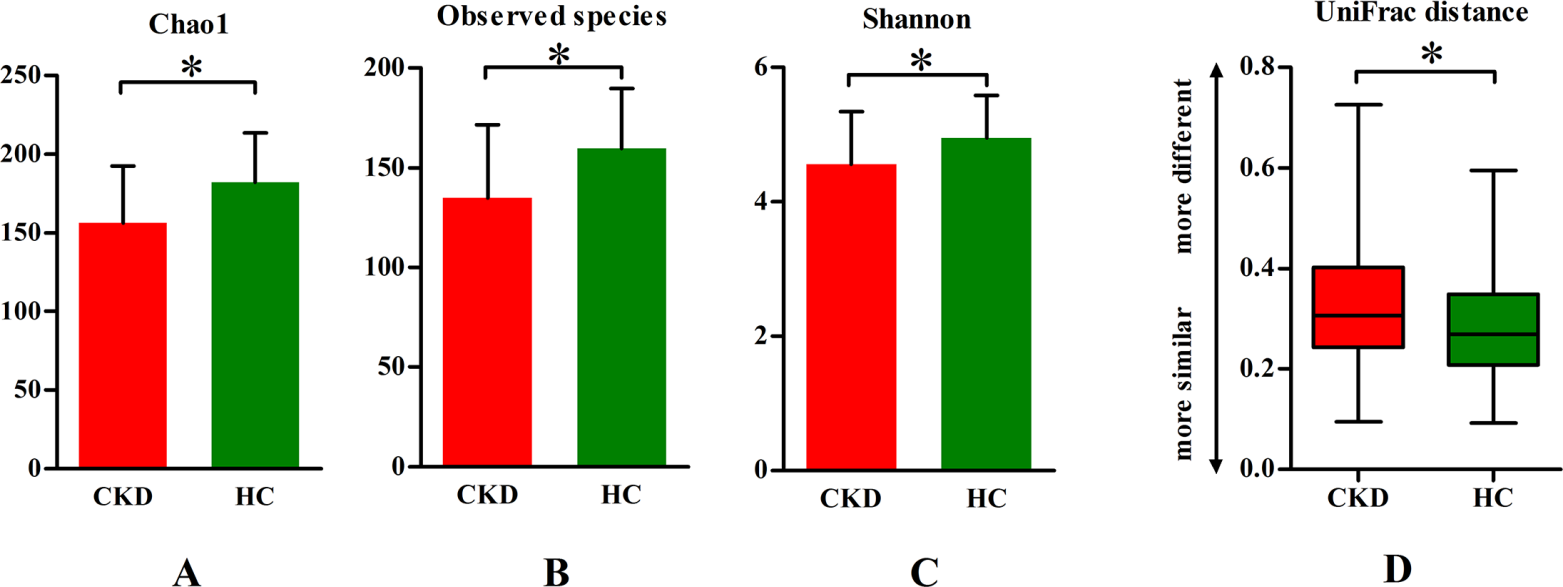
Microbial diversity comparisons between hemodialysis and healthy control group. (A, B, C) Chao1, Observed species and Shannon (α diversity), representing community richness were compared between groups for supragingival plaque. (D) The average weighted UniFrac distance values (β diversity) of supragingival plaque in the two groups. CKD: chronic kidney disease, HC: healthy control. *t*-test or Wilcoxon Rank Sum test, *P < 0.05.

In addition, the α diversity of the supragingival plaques was lower in all the CKD patients than in the control subjects (*P* < 0.05, Fig 1A, 1B and 1C). The heterogeneity of the supragingival plaques in the CKD patients was higher than that in the control subjects based on the weighted UniFrac distance metric. (*P* < 0.05, Fig 1D).

**Fig 2:**
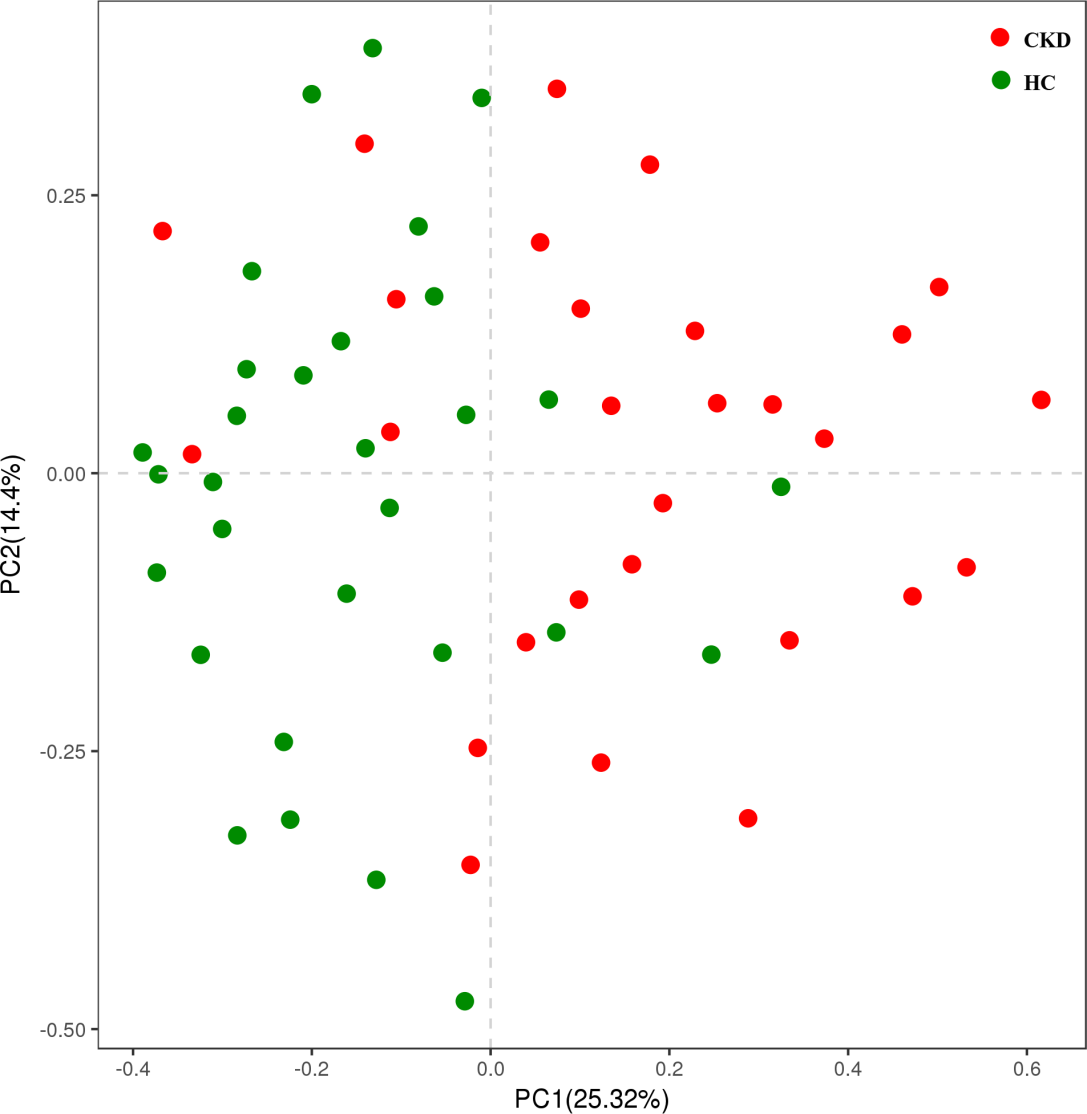
Principal component analysis (PCA) of supragingival plaque based on OTUs with different relative abundances. CKD: chronic kidney disease, HC: healthy control.

The CKD samples were compared with the control samples by PCA based on OTUs with different relative abundance to evaluate the effect of renal failure on the global-scale composition of supragingival bacterial communities (Fig 2). It seemed that the samples had a tendency to cluster into two subgroups, although they were dispersive and both had several samples mixed with each other, indicating a shift in oral microbiome of the CKD patients compared with the healthy controls.

### Caries associated taxa showed higher abundance and prevalence in CKD patients than in healthy controls

**Fig 3:**
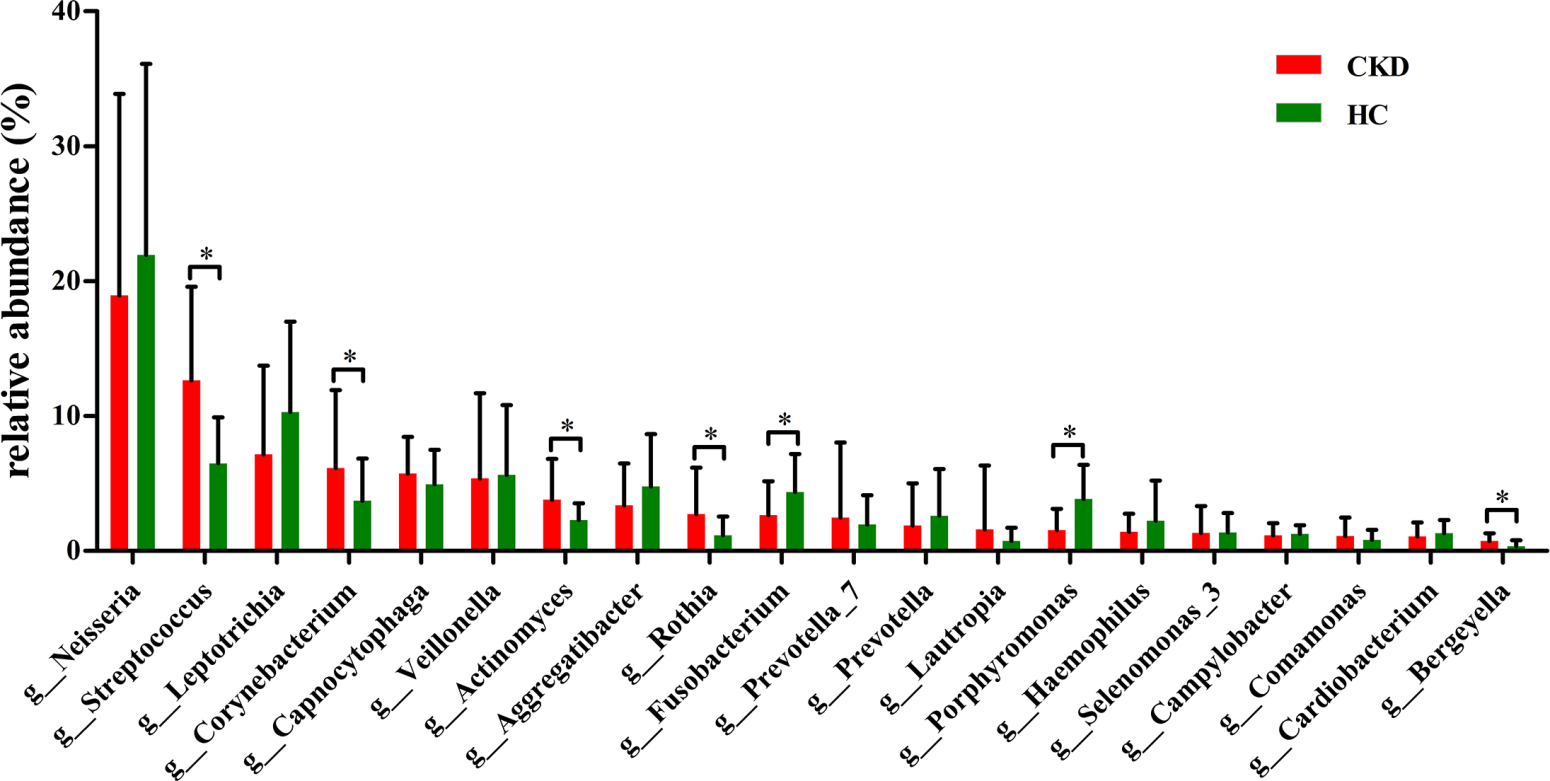
Predominant taxa composition of supragingival plaque at genus level. Predominant genera (top 20) composition of supragingival plaque samples was compared between the two groups. CKD: chronic kidney disease, HC: healthy control. Metastats test, *P < 0.05.

At genus level, the relative abundances of 5 genera (*Streptococcus*, *Corynebacterium*, *Actinomyces*, *Rothia* and *Bergeyella*) were significantly higher in the plaque samples of the CKD patients compared to that of the healthy control (*P* < 0.05, Fig 3). In addition, the genus *Lactobacillus* was also significantly increased, although with relatively low abundance.

**Fig 4:**
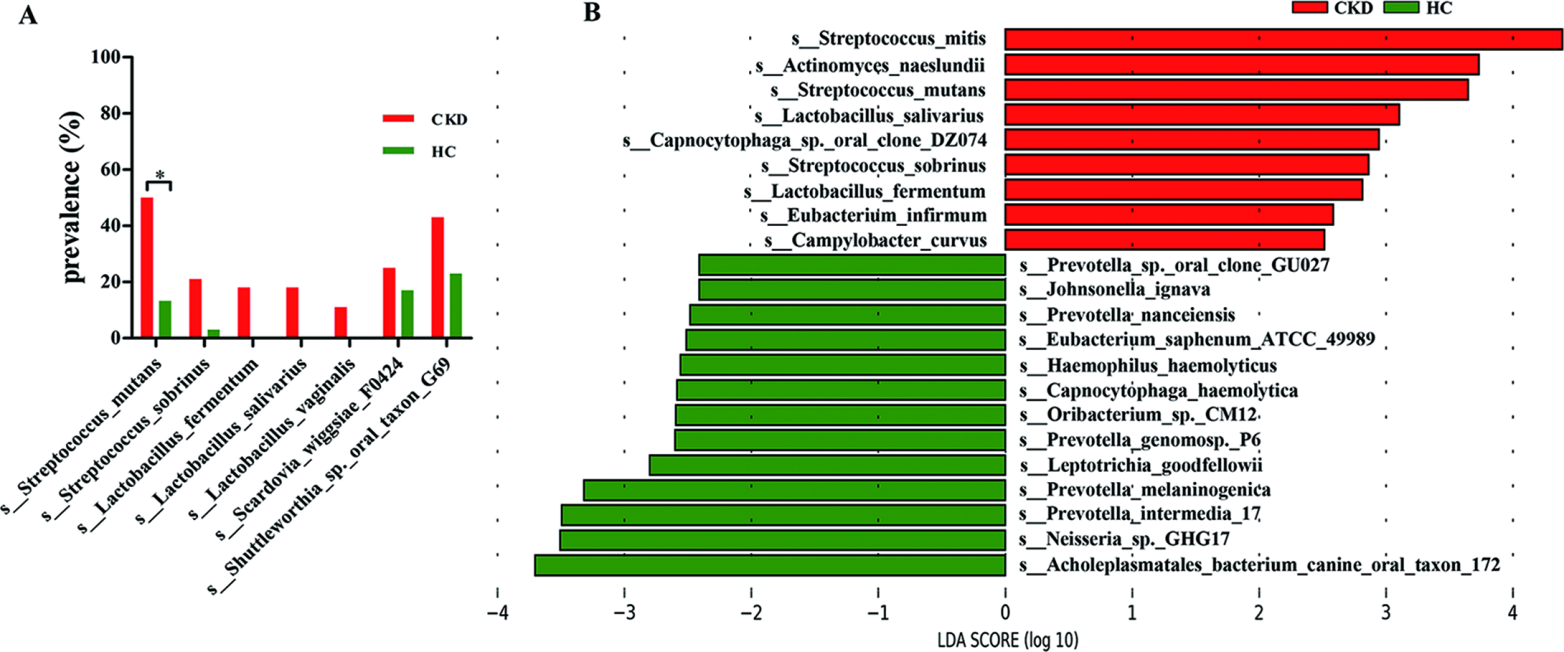
Species with different prevalence or relative abundance. (A) Prevalence of caries related species. (B) Species with different relative abundance based on LEfSe results. CKD: chronic kidney disease, HC: healthy control. Chi-square test, *P < 0.05. LDA score: linear discriminant analysis score, LDA > 2.0.

**Fig 5:**
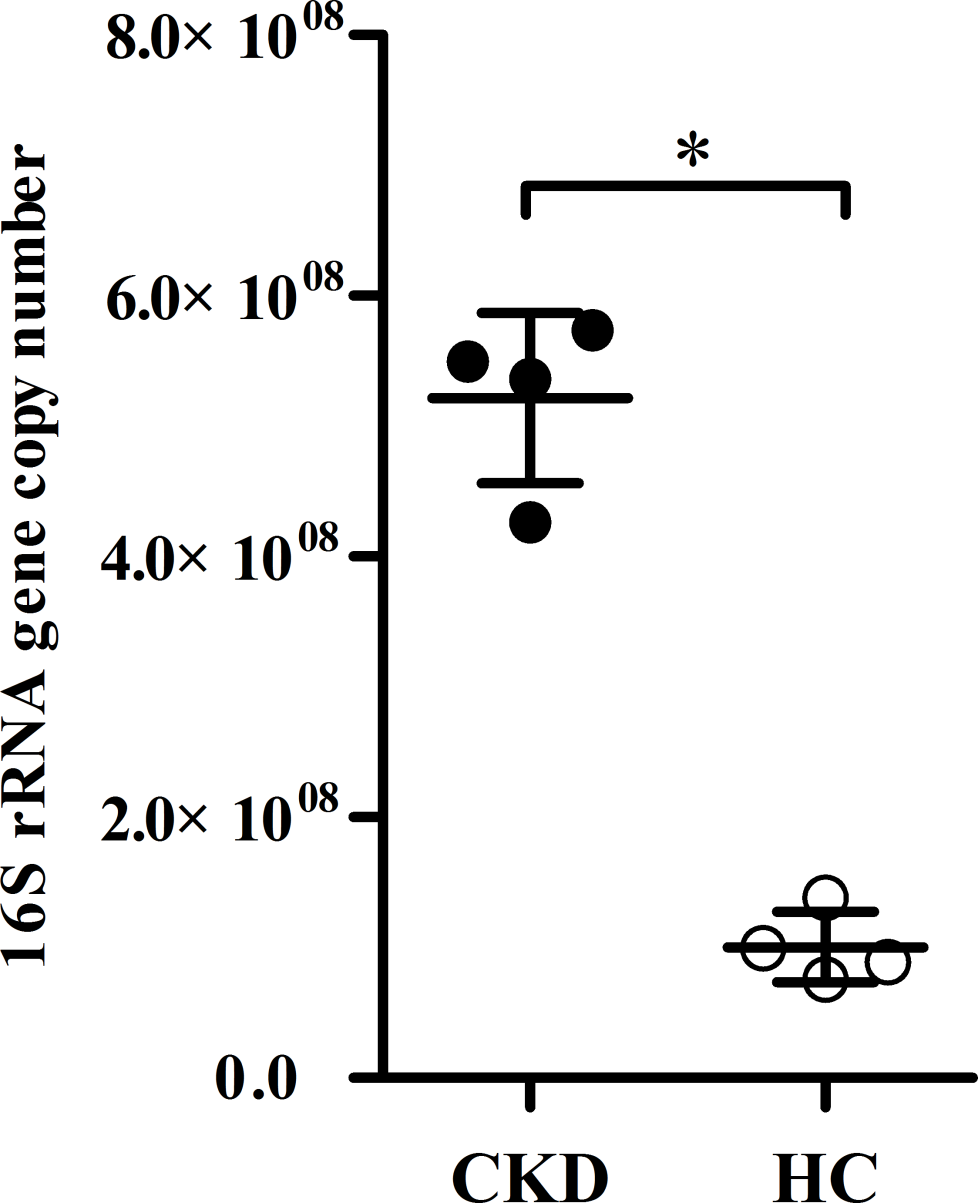
The bacterial load of *Streptococcus mutans* in supragingival plaque, measured by quantitative real-time PCR. CKD: chronic kidney disease, HC: healthy control. Wilcoxon Rank Sum test, *P < 0.05.

As shown in S2 Table and Fig 4, the abundance and prevalence of caries associated species were significantly increased in the CKD group than in the control group, although the relative abundance of these species was mostly in relatively low level. The relative abundance of *Streptococcus mutans*, *Lactobacillus salivarius*, *Lactobacillus fermentum*, *Lactobacillus vaginalis*, *Scardovia wiggsiae F0424*, and *Actinomyces naeslundii* was significantly elevated in the CKD group compared to the control group (*P* < 0.05). *Streptococcus mitis*, the predominant species in the supragingival plaque of the CKD group was also significantly increased compared to the control group (*P* < 0.05). With regard to prevalence, *Streptococcus mutans* were detected more frequently in the CKD group than in control group (*P* < 0.05). The LEfSe analysis also showed that some species with elevated proportions in the CKD patients were associated with caries, such as *Streptococcus mitis*, *Streptococcus mutans*, *Streptococcus sobrinus*, *Lactobacillus fermentum*, *Lactobacillus salivarius*, and *Actinomyces naeslundii* (Fig 4). The higher bacterial load of *Streptococcus mutans* was confirmed in CKD patient by quantitative real-time PCR (*P* < 0.05, Fig 5).

## Discussion

The main results of this study were that the CKD hemodialysis patients showed higher dental caries status than the healthy control subjects, and that the diversity of bacterial community of the supragingival plaque was decreased and the abundance and prevalence of main caries pathogenic species and some caries-related species were also significantly increased in the supragingival plaque of the CKD hemodialysis patients. These results suggested that the CKD hemodialysis patients were more susceptible to dental caries, and therefore, more attentions for caries prevention and treatment should be paid to improve their life quality and even their cardiovascular events or survival.

The CKD hemodialysis patients showed a higher dental caries status. This observation was opposite to our expectation. Some previous studies showed that the hemodialysis patients in children or adults have less dental caries than the control due to the higher concentration of oral urea [7, 8] and show similar dental caries status after kidney transplantation [25]. We also previously observed that rinsing with urea at concentration of 0.25% or above can totally inhibit the sucrose-induced decrease in pH in the dental plaque with a pH telemetry test *in vivo* [26], suggesting that if salivary urea concentration at or more than 0.25% can neutralize pH decrease in the dental plaque induced by dietary carbohydrate intake and can help prevent from dental caries. Therefore, before the present study was performed, we speculated that the CKD hemodialysis patients would have less dental caries due to the facts that they would still have higher concentrations of blood and salivary urea in most time, even having been undergoing hemodialysis for at least one year. The concentrations of blood and salivary urea in the CKD hemodialysis patients were expectedly higher than that in the control subjects. However, the concentration of their salivary urea (20.24 mmol/L = 0.12%) was still too lower (only half of the minimum concentration of 0.25%) to sufficiently neutralize acids in the dental plaques or to prevent from dental caries. The limited inhibitory effect on caries of urea rinsing without calcium or fluoride was also confirmed in our previous study *in situ* [27].

The higher dental caries status of the CKD hemodialysis patients would be the result of many factors. Dental caries is a multifactorial infectious disease, involving cariogenic bacteria acid producing ability, the sugary food intake frequency, calcium concentration in saliva and dental plaque fluid around the tooth, fluoride exposure, and host immunity, etc. Although anti-caries factors, such as the elevated levels of urea, pH, and bicarbonate in the saliva of the CKD patients observed in our or in others’ studies [28, 29], would help prevent from dental caries, they could also be overwhelmed by several other factors which favorite caries, therefore finally leading to the higher caries status of the CKD hemodialysis patients. First, the hemodialysis patients usually have reduced salivary flow rate or xerostomia, because of the hemodialysis treatment, restriction of oral fluid intake, side effects of drug therapy and aging [28, 30, 31]. Saliva plays an important role in protecting the teeth from caries by removing microorganisms, reducing the retention of sugars and neutralizing the acids in the mouth, after carbohydrate consumption. Our results showed that the tendency of feeling dry mouth in CKD patients was also higher than the control group (S1 Table). Second, the lower concentration of salivary calcium in CKD hemodialysis patients would be also an important factor contributing to their higher caries status. Consistent to previous studies [28, 32], we also observed that the concentration of salivary calcium was significantly lower than that of the control subjects. This is probably due to the decrease in formation of 1, 25-dihydroxycholecalciferol and parathyroid hormone metabolism disorder in the end-stage CKD patients [28]. Sufficient calcium is necessary for maintaining a supersaturated condition with respect to enamel of the fluid in contact with it to prevent tooth dissolution [33]. Third, uremic patients usually eat more sugared food than the general population for prevention of hypoglycemia resulting from malnutrition, adrenal insufficiency and dialysis treatment [34]. Similarly, our results of questionnaire (S1 Table) also showed higher frequency of sugary food intake between meals in the CKD patients than in the control subjects. High frequency of consuming sugary snacks or beverages is an important contributor to the development of caries [35].

In the present study, the CKD hemodialysis patients with less than 15 natural teeth were excluded in order to get enough supragingival plaque for microbiome analysis. This could possibly make the number of missing teeth and DMFT level to be relatively lower than that in some previous studies [5, 9]. No difference in FT between the CKD hemodialysis patients and the control subjects might suggest that both groups had a similarly good caries treatment experience. Therefore, the higher caries status in the CKD patients than that the control group was less likely due to the caries treatment experience between the two groups.

The ecological shift in oral microbiome of the CKD hemodialysis patients favorites dental caries. Caries occurs when the ecological balance of oral microbiome was broken by changes in local environmental conditions [36]. Our microbial analysis showed that the acidogenic and aciduric caries related species were increased significantly in the CKD patients than in the control subjects. These species included highly caries-associated pathogenic species, such as *Streptococcus mutans* and *Lactobacillus salivarius*, and some other caries-related aciduric species such as *Lactobacillus fermentum*, *Lactobacillus vaginalis*, *Scardovia wiggsiae F0424*, and *Actinomyces naeslundii*. In addition, the detected predominant species, *Streptococcus mitis*, a potentially acid producers [37], was also increased markedly as compared with the control. The significantly increase of caries related species in the CKD hemodialysis patients might be related to the following reasons. First, frequent sugar intake and poor salivary secretion would result in more frequent and severe pH decreases in the dental plaques, establishing a more acidic environment for the multiplication of these micro-organisms [38, 39]. Second, the CKD hemodialysis patients are usually immunocompromised due to systemic metabolic disorders caused by renal inadequacy [40], which usually disrupts mutual or commensal relationships between microorganism and host [41]. The increase of acidogenic and aciduric caries related species in the CKD hemodialysis patients indicated that the CKD hemodialysis patients were more susceptible to dental caries or needing dental treatment compared to the healthy control subjects. In the present study, we also tried to analyze the species that break down urea, such as *Streptococcus salivarius*. Unfortunately, they were not detectable in the supragingival plaque. The reason for the absence of these species in the supragingival plaque was unknown; it may be due to that their relative abundance was too low to be identified. Therefore, although the salivary urea level and salivary pH in our CKD hemodialysis patients were higher, it still failed to lead to the dominance of urealytic bacteria, a beneficial caries preventive ecology shift.

Cardiovascular disease events are the most common cause of death in the end-stage renal disease [42, 43]. Although periodontitis is associated with the cardiovascular mortality of CKD hemodialysis patients [44, 45], dental caries is also recently suspected to be linked to heart disease, since *Streptococcus mutans*, a species most closely related to caries, is the most frequently detected species in heart valves and aneurysm walls [46]. In addition, a very recent study showed that *Streptococcus mitis*, a predominant species in supragingival plaque and a potential caries related species, is also detected in the atherosclerotic plaques of patients without periodontitis [47]. We observed that the prevalence of *Streptococcus mutans* in the supragingival plaque was significantly higher in the CKD group than in the control group. *Streptococcus mutans* likely enters the bloodstream more readily than the others, and is a possible etiological factor for cardiovascular disease [48]. In addition, there were several other species, possibly related to both caries and endocarditis, such as *Streptococcus mitis* and *Actinomyces naeslundii*, were also increased markedly in the CKD hemodialysis patients. Therefore, the increase of caries-related species in the supragingival plaque might implicate a higher risk for endocarditis in the CKD hemodialysis patients.

The changes in the supragingival plaque microbiota correlated with the dental caries status suggested more attention for developing effective prevention and early intervention strategies for caries of the CKD patients. It is also important for renal physicians to understand the necessity to maintaining dental health for the CKD patients. Greater efforts for caries prevention in the CKD hemodialysis patients than that of general population should be taken to reduce the acidification of oral environment, such as decreasing the sugar intake frequency, improving xerostomia by chewing gum or using artificial saliva to enhance oral self-cleaning ability [49]. Moreover, it is of significance to use fluoridated toothpaste and oral cleaning auxiliary method like dental floss and mouthwash and inhibit the formation of plaque.

The higher *S*. *mutans* in the CKD group than that of the control group was verified by real-time PCR. However, the verification of *S*. *mutans* by real-time PCR was only performed for all the four *S*. *mutans* detectable samples in the control group and four randomly selected *S*. *mutans* detectable samples in the CKD group, but not all the *S*. *mutans* detectable samples in the CKD group. This could be one of the limitations of the present study. The other limitation could be that the exclusion criteria for the patients in terms of using antibiotics was not based on three months or longer, but only one month, due to the reasons that the end stage CKD patients are usually susceptible to infection and therefore it was difficult to recruit the end stage CKD patients without antibiotics treatment within or more than 3 months.

In conclusion, oral environment in the CKD hemodialysis patients appeared in favor of the growth of cariogenic bacteria leading to elevated caries risk, and even possibly increase in the cardiovascular death risk. Therefore, detecting and management of caries related microbial shifts at earlier and reversible stage to maintain dental health is important for improving quality of life, cardiovascular events and survival in CKD hemodialysis patients.

## Supporting information

S1 Fig Rarefaction curves of supragingival plaque samples. Most curves become flat in the end indicating that a reasonable number of tags were analyzed.
(TIF)

S2 Fig Microbial composition of supragingival plaque samples at phylum level. (TIF)

S1 Table Comparison of oral behaviors between groups. CKD: chronic kidney disease, HC: healthy control, Chi-square test, * *P* < 0.05
(DOCX)

S2 Table Relative abundance and prevalence of caries-related species significantly higher in the CKD hemodialysis group than in the healthy control group. Metaststs test or Chi-square test, *P < 0.05.
(DOCX)
