# Supplementary figures and images for: Carious status and supragingival plaque microbiota in hemodialysis patients
Source: PLoS One. 2018 Oct 9;13(10):e0204674. doi: 10.1371/journal.pone.0204674 (PMC6177147; doi:10.1371/journal.pone.0204674)

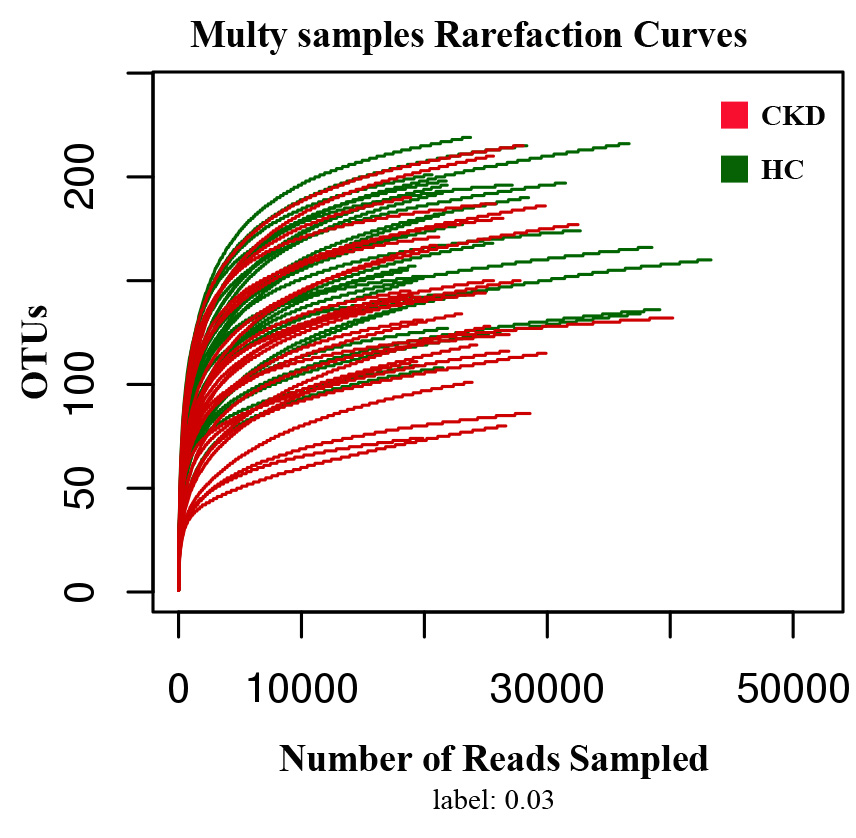

Supplement: S1 Fig — Most curves become flat in the end indicating that a reasonable number of tags were analyzed. (TIF) [file pone.0204674.s001.tif]

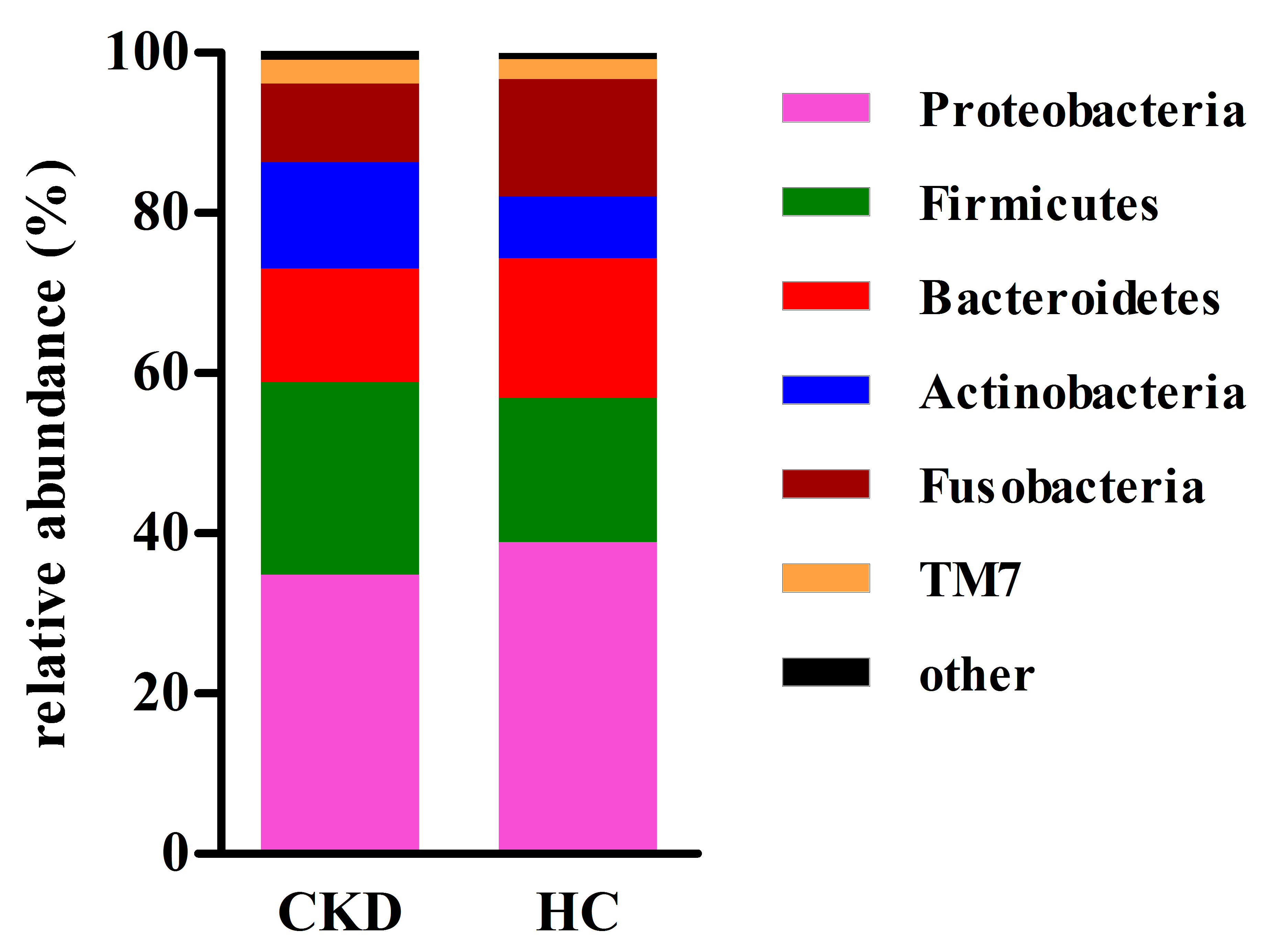

Supplement: S2 Fig — (TIF) [file pone.0204674.s002.tif]
