# Supplementary material for: Carious status and supragingival plaque microbiota in hemodialysis patients
Source: PLoS One. 2018 Oct 9;13(10):e0204674. doi: 10.1371/journal.pone.0204674 (PMC6177147; doi:10.1371/journal.pone.0204674)
Supplement: S1 Table — CKD: chronic kidney disease, HC: healthy control, Chi-square test, * P < 0.05 (DOCX) [file pone.0204674.s003.docx]

**S1 Table Comparison of oral behaviors between groups.**

| Questions | CKD  % (n) | HC  % (n) |
| --- | --- | --- |
| Sugary snacks/beverages taking frequency*  Never  Once or twice  Three or four times  Five times or more | 37.0% (10)  11.2% (3)  37.0% (10)  14.8% (4) | 96.4% (27)  3.6% (1)  0.0% (0)  0.0% (0) |
| Brushing teeth  never  <1×/wk  <1×/d  1×/d  ≥2×/d | 0.0% (0)  0.0% (0)  0.0% (0)  13.8% (4)  86.2% (25) | 0.0% (0)  0.0% (0)  0.0% (0)  30.0% (9)  70.0% (21) |
| Using toothpick  never  <1×/wk  <1×/d  1×/d  ≥2×/d | 58.6% (17)  3.4% (1)  17.2% (5)  13.8% (4)  6.9% (2) | 56.7% (17)  10.0% (3)  13.3% (4)  3.3% (1)  16.7% (5) |
| Using dental floss  never  <1×/wk  <1×/d  1×/d  ≥2×/d | 93.1% (27)  3.4% (1)  0.0% (0)  3.4% (1)  0.0% (0) | 83.3% (25)  6.7% (2)  3.3% (1)  0.0% (0)  6.7% (2) |
| Using mouthwash  never  <1×/wk  <1×/d  1×/d  ≥2×/d | 86.2% (25)  6.9% (2)  3.4% (1)  0.0% (0)  3.4% (1) | 76.7% (23)  13.3% (4)  6.7% (2)  3.3% (1)  0.0% (0) |
| Xerostomia*  Never  Occasionally  Sometimes  Always | 10.3% (3)  17.2% (5)  44.8% (13)  27.6% (8) | 16.7% (5)  43.3% (13)  26.7% (8)  13.3% (4) |

CKD: chronic kidney disease, HC: healthy control, Chi-square test, * *P* < 0.05
