# Supplementary material for: Carious status and supragingival plaque microbiota in hemodialysis patients
Source: PLoS One. 2018 Oct 9;13(10):e0204674. doi: 10.1371/journal.pone.0204674 (PMC6177147; doi:10.1371/journal.pone.0204674)
Supplement: S2 Table — Metaststs test or Chi-square test, *P < 0.05. (DOCX) [file pone.0204674.s004.docx]

**S2 Table** **Relative abundance and prevalence of caries-related species significantly higher in the CKD hemodialysis group than in the healthy control group.**

| **Species** | **Abundance** | **Prevalence** | **Opinions** | **Reference** |
| --- | --- | --- | --- | --- |
| *Streptococcus mutans* | higher^*^ | higher^*^ | caries related;  CVD related | [1, 2] |
| *Lactobacillus salivarius* | higher^*^ | higher | caries related | [3] |
| *Lactobacillus fermentum* | higher^*^ | higher | caries related | [4] |
| *Lactobacillus vaginalis* | higher^*^ | higher | caries related | [5] |
| *Scardovia wiggsiae F0424* | higher^*^ | higher | caries related | [6] |
| *Streptococcus mitis* | higher^*^ | equal | potential caries related;  CVD related | [7, 8] |
| *Actinomyces naeslundii* | higher^*^ | equal | root caries related;  endocarditis related | [9, 10] |
| *Streptococcus sobrinus* | higher | higher | caries related | [1] |
| *Shuttleworthia sp. oral taxon G69* | higher | higher | dentin caries related | [11] |

CVD: cardiovascular disease, Metaststs test or Chi-square test, ^*^*P* < 0.05.

**Reference**

1. Corby PM, Lyons-Weiler J, Bretz WA, Hart TC, Aas JA, Boumenna T, et al. Microbial risk indicators of early childhood caries. J Clin Microbiol. 2005;43:5753-9. doi: 10.1128/JCM.43.11.5753-5759.2005. PubMed PMID: 16272513; PubMed Central PMCID: PMCPMC1287835.

2. Nagata E, Oho T. Invasive Streptococcus mutans induces inflammatory cytokine production in human aortic endothelial cells via regulation of intracellular toll-like receptor 2 and nucleotide-binding oligomerization domain 2. Mol Oral Microbiol. 2017;32:131-41. doi: 10.1111/omi.12159. PubMed PMID: 27004566.

3. Richards VP, Alvarez AJ, Luce AR, Bedenbaugh M, Mitchell ML, Burne RA, et al. Microbiomes of Site-Specific Dental Plaques from Children with Different Caries Status. Infect Immun. 2017;85:e00106-17. doi: 10.1128/IAI.00106-17. PubMed PMID: 28507066; PubMed Central PMCID: PMCPMC5520424.

4. Mitrakul K, Vongsavan K, Suratanachaikul P. Prevalence of Streptococcus mutans and Lactobacillus fermentum and their association with caries and dietary habits in preschool Thai children. Eur Arch Paediatr Dent. 2013;14:83-7. doi: 10.1007/s40368-013-0017-8. PubMed PMID: 23576064.

5. Kanasi E, Johansson I, Lu SC, Kressin NR, Nunn ME, Kent R, Jr., et al. Microbial risk markers for childhood caries in pediatricians' offices. J Dent Res. 2010;89:378-83. doi: 10.1177/0022034509360010. PubMed PMID: 20164496; PubMed Central PMCID: PMCPMC2880172.

6. Row L, Repp MR, Kingsley K. Screening of a Pediatric and Adult Clinic Population for Caries Pathogen Scardovia Wiggsiae. J Clin Pediatr Dent. 2016;40:438-44. doi: 10.17796/1053-4628-40.6.438. PubMed PMID: 27805882.

7. Aas JA, Griffen AL, Dardis SR, Lee AM, Olsen I, Dewhirst FE, et al. Bacteria of dental caries in primary and permanent teeth in children and young adults. J Clin Microbiol. 2008;46:1407-17. doi: 10.1128/JCM.01410-07. PubMed PMID: 18216213; PubMed Central PMCID: PMCPMC2292933.

8. Eberhard J, Stumpp N, Winkel A, Schrimpf C, Bisdas T, Orzak P, et al. Streptococcus mitis and Gemella haemolysans were simultaneously found in atherosclerotic and oral plaques of elderly without periodontitis-a pilot study. Clin Oral Investig. 2017;21:447-52. doi: 10.1007/s00784-016-1811-6. PubMed PMID: WOS:000391388300049.

9. Dame-Teixeira N, Parolo CCF, Maltz M, Tugnait A, Devine D, Do T. Actinomyces spp. gene expression in root caries lesions. J Oral Microbiol. 2016;8:1-13. doi: 10.3402/jom.v8.32383. PubMed PMID: WOS:000389976800001.

10. Cortes CD, Urban C, Turett G. Actinomyces naeslundii: An Uncommon Cause of Endocarditis. Case Rep Infect Dis. 2015;2015:1-4. doi: 10.1155/2015/602462. PubMed PMID: WOS:000215185700047.

11. Jiang W, Ling ZX, Lin XL, Chen YD, Zhang J, Yu JJ, et al. Pyrosequencing Analysis of Oral Microbiota Shifting in Various Caries States in Childhood. Microb Ecol. 2014;67:962-9. doi: 10.1007/s00248-014-0372-y. PubMed PMID: WOS:000334495000023.
